# Supplementary material for: 1D versus 2D cocrystals growth via microspacing in-air sublimation
Source: Nat Commun. 2019 Feb 15;10:761. doi: 10.1038/s41467-019-08712-1 (PMC6377649; doi:10.1038/s41467-019-08712-1)
Supplement: Supplementary file 1 — Supplementary Information [file 41467_2019_8712_MOESM1_ESM.pdf]

Supplementary Information

1D Versus 2D Cocrystals Growth via Microspacing In-Air Sublimation

Ye et al.

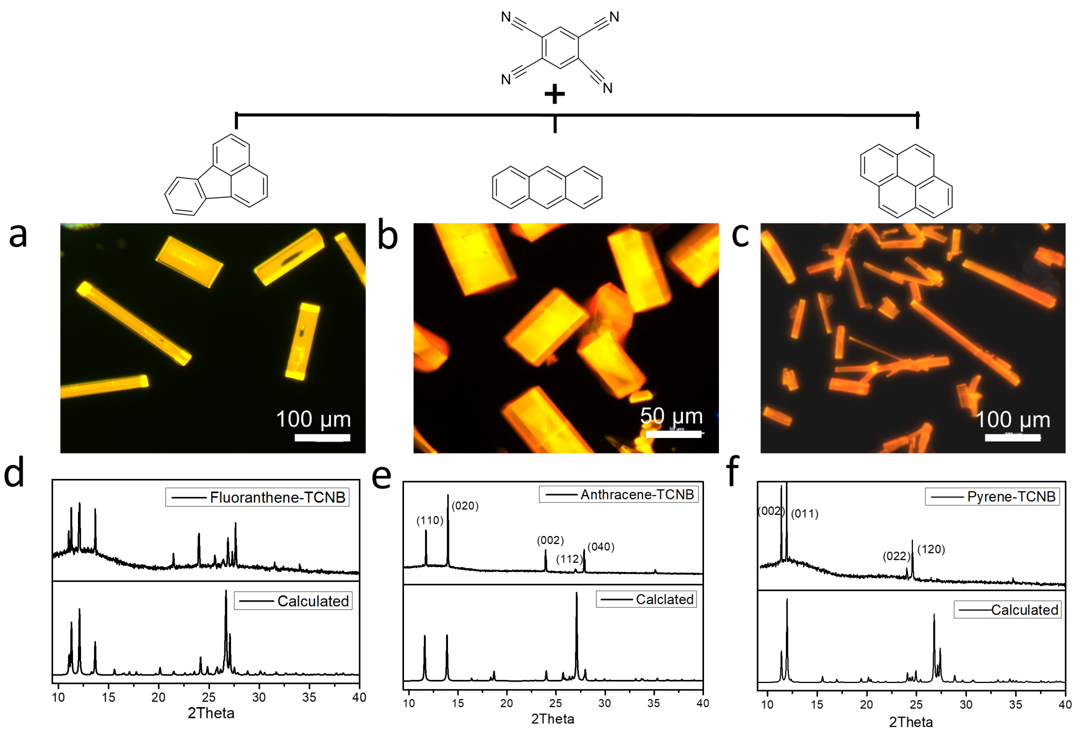

Supplementary Figure 1 (a-c) Fluorescence microscope images of fluoranthene-TCNB, anthracene-TCNB, and pyrene-TCNB cocrystals grown by MAS. (d-e) The corresponding XRD patterns of cocrystals.

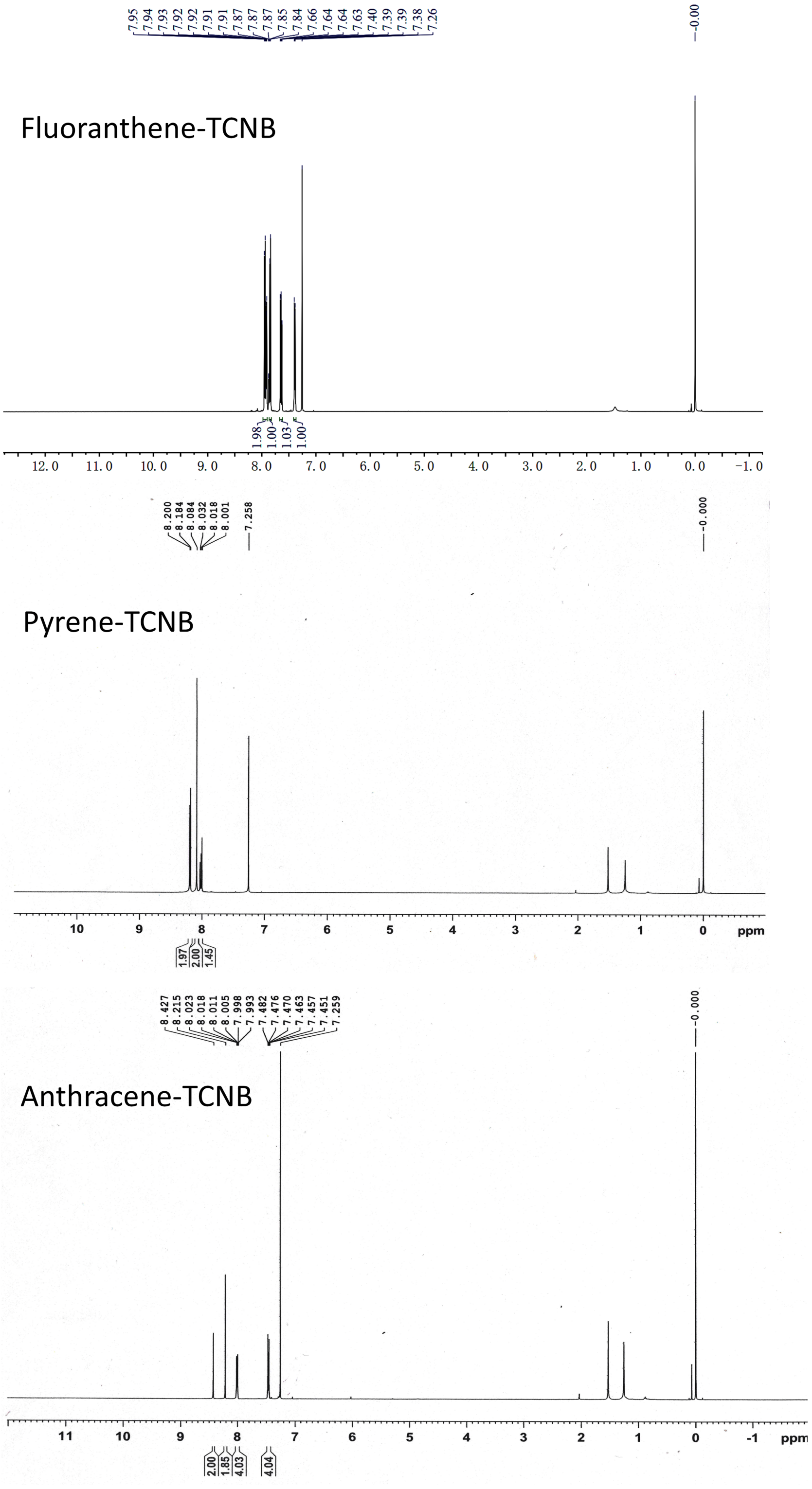

Supplementary Figure 2 ^{1}H NMR spectra of fluoranthene-TCNB, pyrene-TCNB and anthracene-TCNB cocrystals grown by MAS.

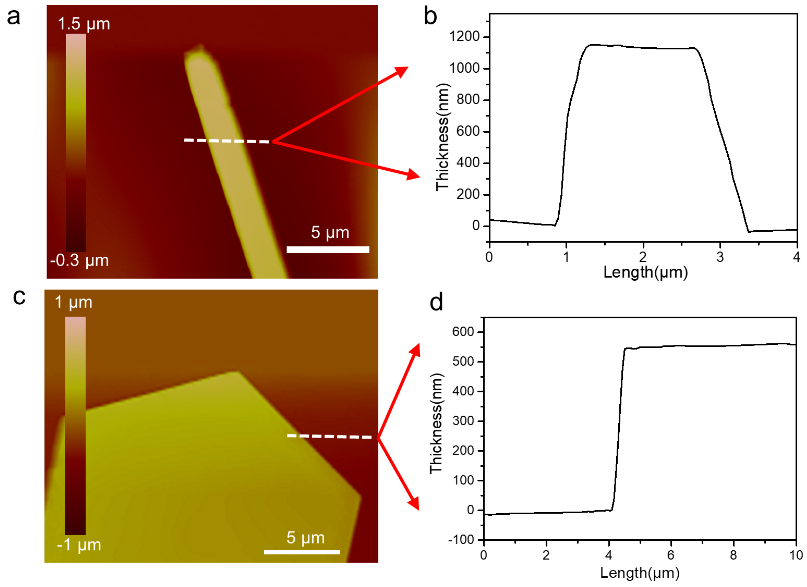

Supplementary Figure 3 AFM image of thin MAS-grown 1D needle-like (a) and 2D plated (c) FTCs cocrystal. (b and d) The corresponding thickness profile measured along dashed white line.

Supplementary Table 1. Crystallographic data and structure refinement parameters of FTCs.

Compound reference	Fluoranthene-TCNB
Formula Mass	380.40
Crystal system	Orthorhombic
Space group	P21/c
a/Å	7.362(5)
b/Å	8.263(6)
c/Å	31.29(2)
α, γ (deg)	90
B (deg)	90.250(14)
Unit cell volume/Å^3	1903(2)
Temperature/K	293
No. of formula units per unit cell, Z	4
Collected reflns	17970
Unique reflns	3348
R_{int}	0.0311
Final R_1 values (I > 2σ(I))	0.0364
Final $wR(F^2)$ values (I > 2σ(I))	0.0932
Final $wR(F^2)$ values (all data)	0.1004
Goodness of fit on F^2	1.043

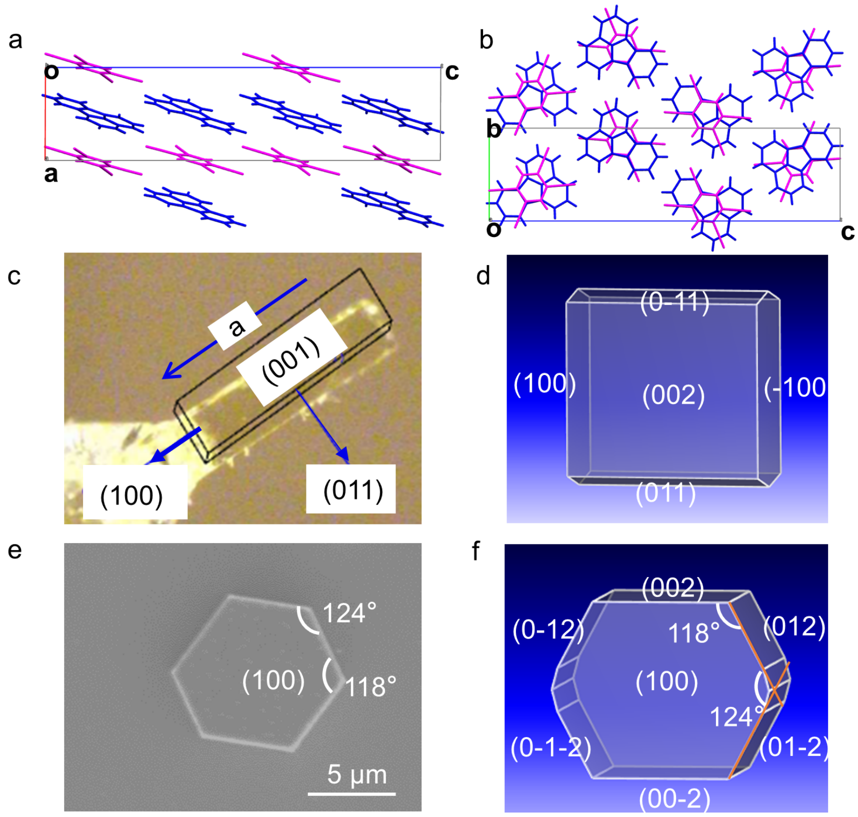

Supplementary Figure 4 Molecular packing of FTCs in the basal (a-c) plane (a) and (b-c) plane (b). (c) Face-indexing graphics of 1D needle-like FTCs. (d) Predicted crystal morphology based on the attachment energies by using the material studio package. (e) SEM image of 2D plate-like FTCs. (f) The equilibrium shape for minimum total surface energy, calculated by the software of Materials Studio package.

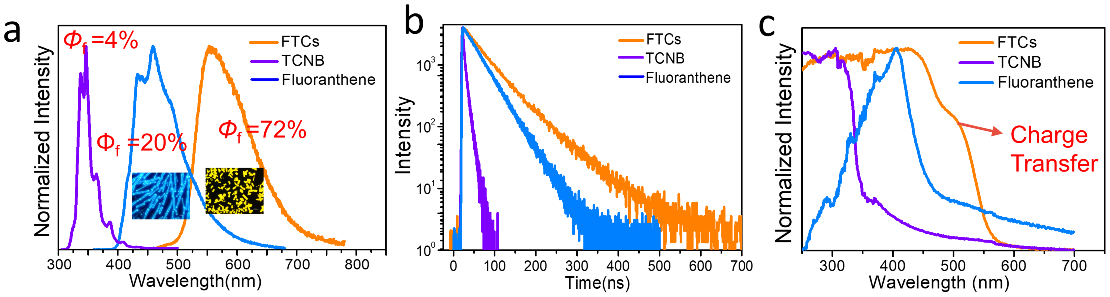

Supplementary Figure 5 Luminescent properties of grown cocrystals. The PL spectra (a) and absorption spectra (b) of as-grown fluoranthene, TCNB and FTCs, (inset: fluorescence microscopy images of fluoranthene and cocrystals grown by MAS) (c) Time-resolved fluorescence decay profile of fluoranthene, TCNB, and FTCs.

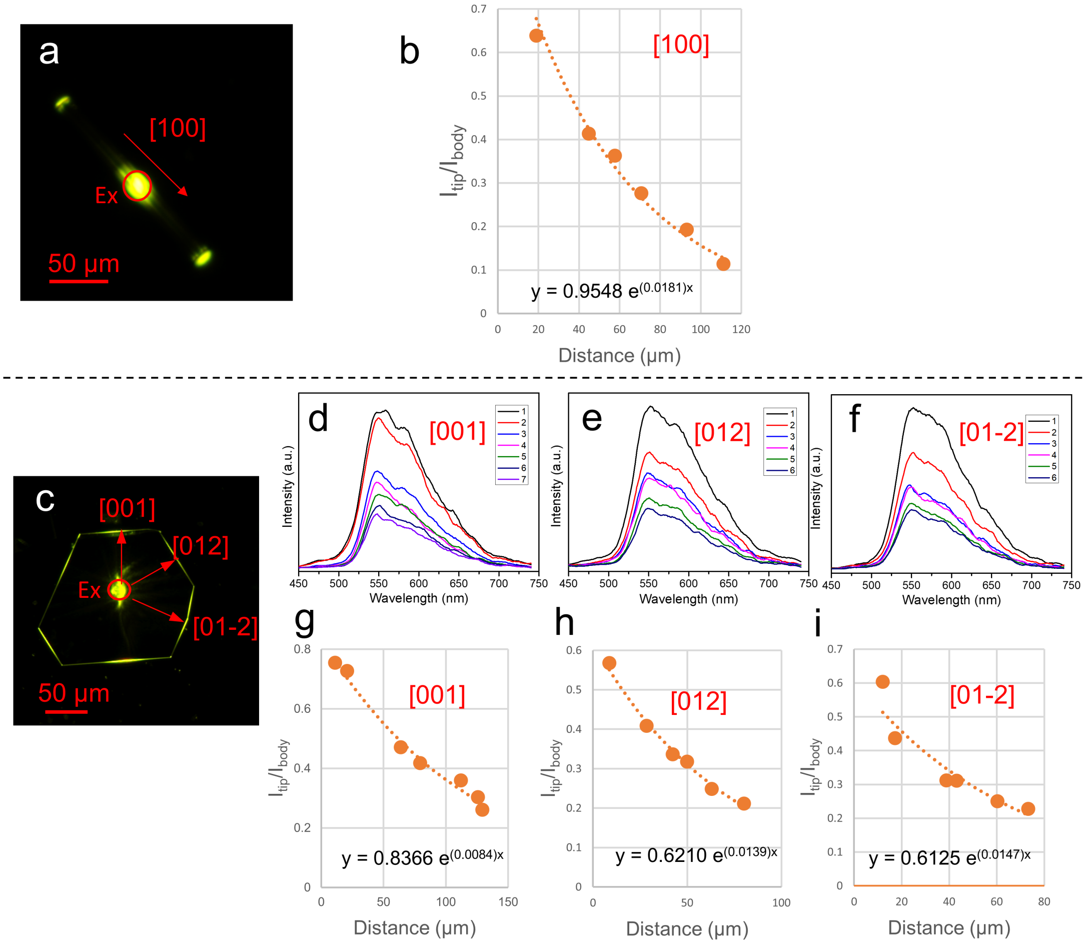

Supplementary Figure 6 (a and c) Fluorescence microscopy images 1D and 2D FTCs. (b, g-i) Ratio of PL intensity, I_{tip}/I_{body}, against the propagation distance of different directions. The curves were fitted by an exponential decay function. (d-e) μ-PL spectra collected from the tip of FTCs when the excitation spot was moved.

The PL intensity at the excited site along the body of 1D or 2D FTC (I_{body}) and at the emitting edge (I_{tip}) were recorded, and the ratio I_{tip}/I_{body} shows a single-exponential decay against propagation distance, which indicates the active nature of the optical waveguide. The optical-loss coefficient (R) was calculated by single-exponential fitting $I_{tip}/I_{body} = A\ e^{-RD}$, where D is the distance between the excited site and the emitting edge.

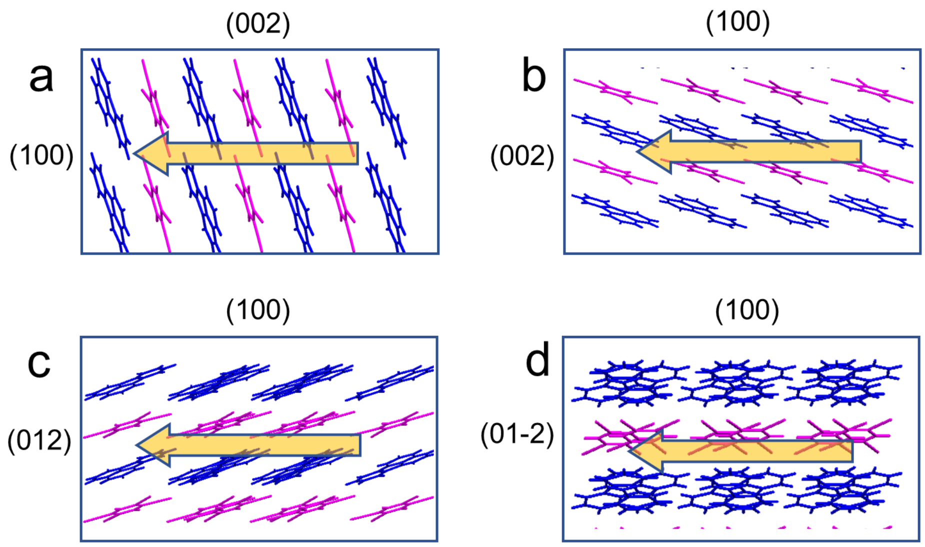

Supplementary Figure 7 (a) Molecular packing of the 1D FTCs along the [100] direction. (b-d) Molecular packing of 2D FTCs along the [002], [012] and [01-2] direction.

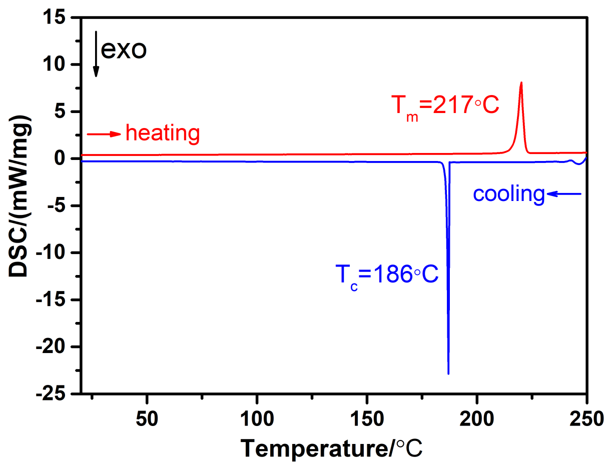

Supplementary Figure 8 DSC analyses of FTCs at a rate of 10°C min^{-1}. The red curve is the heating curve and the blue one is the cooling curve. The T_c and T_m denote the recrystallization and melting temperatures respectively.

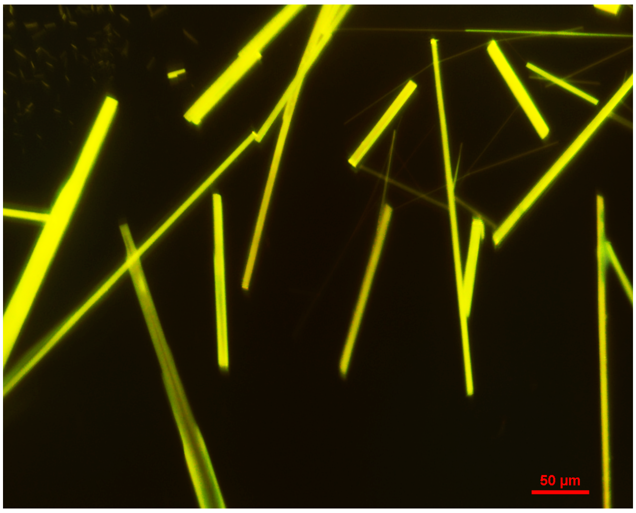

Supplementary Figure 9 Fluorescence microscope image of FTCs grown from solution.

Supplementary Table 2. Surface energies of various crystal facets (hkl) calculated by the material studio package.

**hkl**	**(002)**	**(011)**	**(100)**	**(012)**
γ (kcal·mol^{-1})	8.02	29.38	43.49	30.00

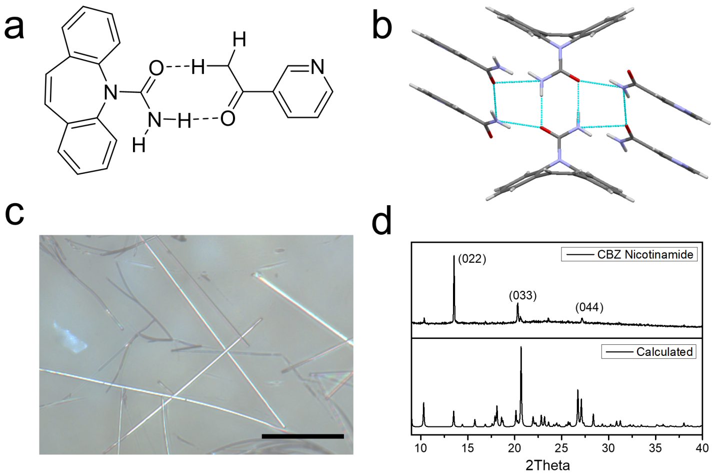

Supplementary Figure 10 Chemical structures (a), single crystal structures (CCDC NO.: 212375) (b), microscope image (c) and XRD patterns (d) of carbamazepine-nicotinamide cocrystals grown by MAS.

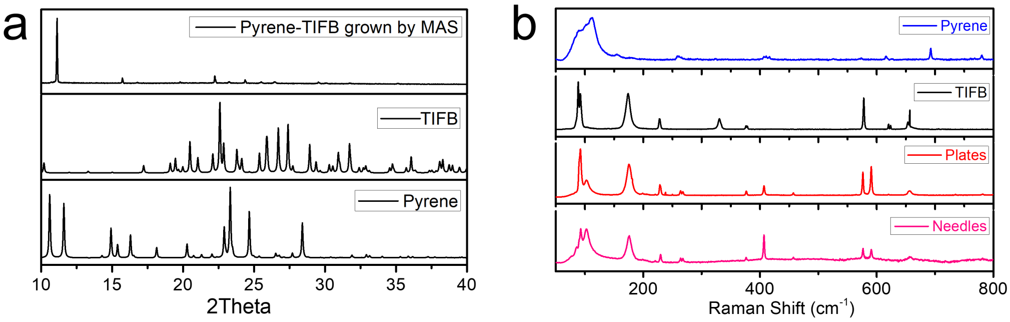

Supplementary Figure 11 XRD pattern (a) and Raman spectra (b) of 1D needle-like and 2D plate-like pyrene–TIFB cocrystals, TIFB and pyrene.

The XRD patterns and Raman spectra of the as-grown pyrene–TIFB cocrystals are distinct from that of each constituent molecule, suggesting the formation of cocrystals. The difference of relative peak intensity in Raman spectra of the needle-like and plate-like cocrystals may be related with different morphology.

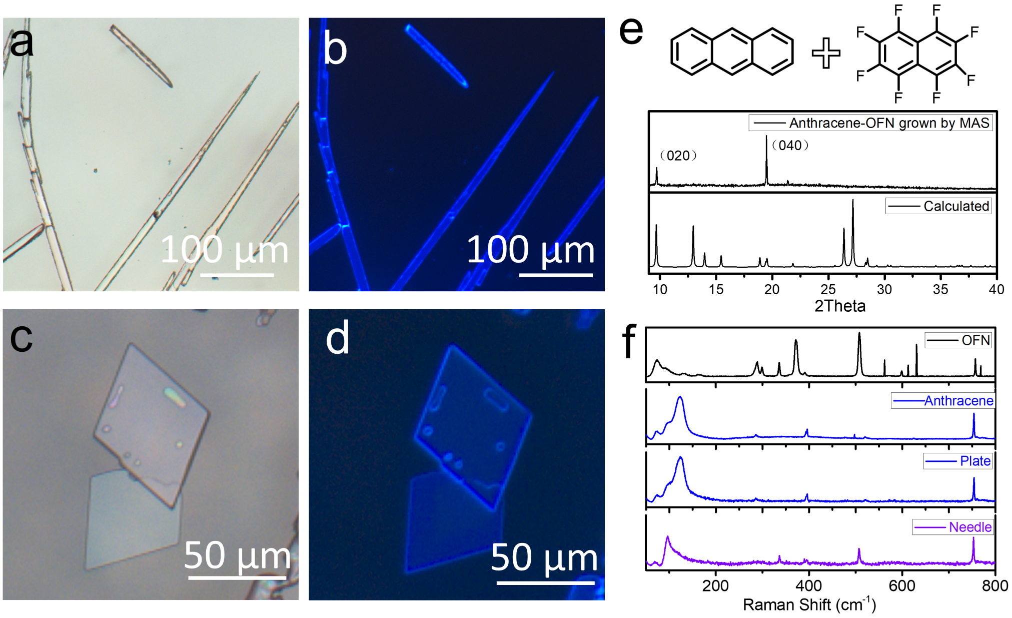

Supplementary Figure 12 Microscope images (a, c) and fluorescence microscope images (b, d), XRD pattern (e) and Raman spectra (f) of 1D needle-like anthracene-OFN cocrystals (a-b) and 2D plate-like (c-d) anthracene crystals. (Scale bar: 100 µm for a and b, 50 µm for others).

For the anthracene–octafluoronaphthalene complex, when growing at relative lower temperature (100 °C), 1D needles crystalized on the substrate (Supplementary Figure 12a); when growing at relative higher temperature (higher than 120 °C), 2D plates formed (Supplementary Figure 12c). However, it is not the case that morphology control is realized on anthracene–OFN. According to the XRD pattern and Raman spectra in **Supplementary Figure 12e–f**, the 1D needles are indeed cocrystals, with oriented growth of b-axis perpendicular to the substrate surface. But the Raman spectra of 2D plates shows that they actually are anthracene crystals.

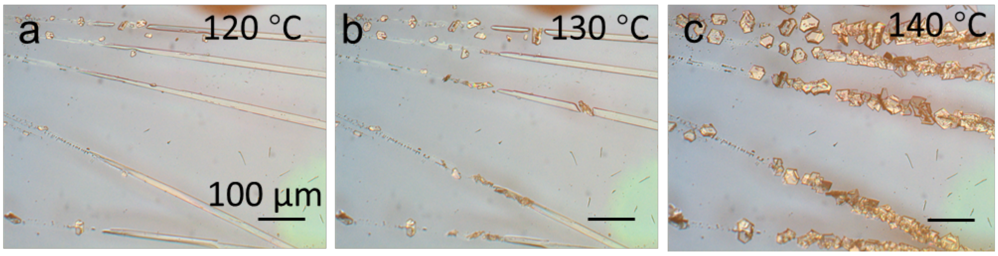

Supplementary Figure 13 Microscope images of in situ observation of transformation from needle-like cocrystals to plate-like anthracene crystals during the heating process. (Scale bar: 100 µm). During the heating process the needle-like cocrystals decomposed and then plated anthracene crystals appeared.

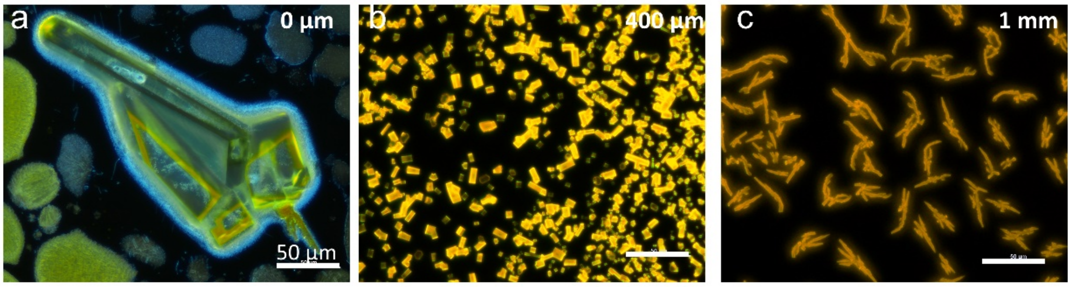

Supplementary Figure 14 Fluorescence microscope images of cocrystals grown by MAS with different space h. (a) The top substrate is prone to directly contact with the melt, so we label it as 0 µm, cocrystal is hard to crystalize from the melt, resulting many glassy state solidified residuals. (b) When the space h is of about 400 µm, the crystallinity is okay, but maybe because of too many of nucleuses the length of the grown cocrystals is much shorter than that in a case of 150 µm. (c) For the case of an even larger h of 1 mm, the grown cocrystals are no more isolated single crystals but dendritic polycrystals.
